# Mineral Composition and Consumer Acceptability of Amaranthus Leaf Powder Supplemented *Ujeqe* for Improved Nutrition Security

**DOI:** 10.3390/foods12112182

**Published:** 2023-05-29

**Authors:** Ruth N. Olusanya, Unathi Kolanisi, Nomali Z. Ngobese

**Affiliations:** 1 Discipline of Food Security, School of Agricultural, Earth and Environmental Science, University of KwaZulu-Natal, Scottsville, Pietermaritzburg 3209, South Africa; 2 Department of Consumer Science, University of Zululand, 24 Main Road, Kwadlangezwa, uThungulu 3886, South Africa; 3 Unit for Environmental Sciences and Management, Faculty of Natural and Agricultural Sciences, North-West University, Private Bag X6001, Potchefstroom 2520, South Africa

**Keywords:** *Amaranthus dubius* leaves, nutrient enhancement, nutrition security, Zulu bread, sensory evaluation

## Abstract

Malnutrition, especially micronutrient deficiency, is a widespread health challenge that predominantly affects young children, young ladies who are within the productive age, refugees, and older adults who reside in rural communities and informal settlements in underdeveloped and developing countries. Malnutrition is caused by consuming either too little or too much of one or more food nutrients. Additionally, monotonous dietary lifestyle, especially the over-reliance on staple foods, is identified among the top factors limiting many individuals’ intake of essential nutrients. Thus, enriching starchy and cereal-based staple foods including *Ujeqe* (steamed bread) with fruits and especially leafy vegetables is being suggested as a strategic medium for essential nutrient delivery to malnourished populations and especially the *Ujeqe* regular consumers. Amaranthus, called pigweed, has been rediscovered as a nutrient-dense multipurpose plant. The seed has been explored as a nutrient-enhancer in staple foods; however, the leaves are underutilized, especially in *Ujeqe*. This study aims to enhance the mineral content of *Ujeqe*. An integrated research approach was used where *Amaranthus dubius* was self-processed into leaf powder. Amaranthus leaf powder (ALP), and the ALP-supplemented wheat flour *Ujeqe* prototypes 0%, 2%, 4%, and 6% were investigated for their mineral composition. Sensory evaluations of enriched *Ujeqe* were conducted using 60 panelists on a five-point hedonic scale. Findings show that the moisture contents of the raw materials and the supplemented prototypes were low, indicating a good shelf life of the food ingredient before being used for *Ujeqe* development. Carbohydrates of raw materials ranged from 41.6–74.3%, fat ranged from 1.58–4.47%, ash ranged from 2.37–17.97%, and protein ranged from 11.96–31.56%. Additionally, fat, protein, and ash content had significant differences at (*p* < 0.05). The moisture content of enhanced *Ujeqe* was equally low, connoting keeping quality of the sample. The increase concentration of ALP led to an enriched *Ujeqe* especially in the ash and protein content. Similarly, calcium, copper, potassium, phosphorus, manganese, and iron content were significantly influenced at (*p* < 0.05); 2% ALP-supplemented *Ujeqe* was the most acceptable prototype as the control sample, 6% was the least preferred prototype. Although ALP *dubius* can enrich staple foods including (*Ujeqe*), this study declared that higher addition of ALP *dubius* leads to low consumer acceptability rate of the *Ujeqe,* which is not statistically significant. Amaranthus is an economical source of fiber, which was not investigated in the study. Therefore, further studies can explore the fiber content of the ALP-supplemented *Ujeqe*.

## 1. Introduction

Worldwide, people eat a lot of varieties of cereal-based meals as staples, particularly wheat-based foods that include *Ujeqe* (steamed bread) [1]. Wheat flour is the major ingredient for bread production, including *Ujeqe*, which may contain a wide range of nutrients, especially carbohydrates, for human functioning [2,3]. However, refined wheat-based foods lack dietary fiber and other essential macro and micronutrients; thus, wheat is primarily considered as a good energy source with limited essential nutrients [1,2,3]. This underpinned the reason behind the current, increased consumer demand for healthy and nutritious foods worldwide. Therefore, people’s need for healthy and nutritious food can still be attained via the people’s food of preferences but through nutrient enrichment of staple foods. Hence, tackling malnutrition has led to the increased interest in studies exploring available nutrient-dense foods that are underutilized, including functional food materials such as *Amaranthus* leaves. Hence, most studies are geared towards adding value to staple food to tackle the world’s food and nutrition insecurity, which are pandemic among malnourished populations living in rural settings or disadvantaged communities across the world. The motivation of this studies revolves around the demand from the United Nations (UN), which set some sustainable development goals that the human population must pursue [4]. Among the 17 goals set for agenda 2030 include (1) production, (2) adequate consumption, and (3) human wellbeing and good health, which are to be pursued as goals, respectively [5]. In this regard, the division between economic growth and environmental depletion and the need to enhance efficiency in resources that will promote sustainable lifestyles, especially people’s dietary lifestyles, is hereby credited to be the starting point and focus of any eco-friendly consumption and production of food [5]. Previous studies show that cereals have been investigated as sources of staple foods. Wheat, being a cereal, has been used to produce several wheat-based food products and has been made from plain flours and a composite flour to make foods, including a variety of different white bread similar to *Ujeqe*, as the most widely consumed food in many regions of the world including South Africa [6,7,8]. Inadequate consumption of foods that are more of quantity and not quality based; a monotonous diet of cereals and starch-based foods has been identified as a factor contributing to malnutrition, which is identified as a public health challenge that is preventable and curative if a conscious and sustainable intake of diversified available nutrient dense foods can be intentionally consumed. The consumption of vegetables, especially the indigenous leafy greens, is low even in South Africa; hence, their potential to provide food and nutrition security to the most vulnerable groups has been underutilized. Predominant malnutrition still affects young children, young ladies who are within the productive age, refugees, and older adults living mostly in rural communities and informal settlements in underdeveloped and developing countries [9,10]. *Ujeqe* is a food product made from refined wheat- that is well appreciated by all South Africans excluding babies. It is precisely appreciated among the Zulu ethnic group [11]. *Ujeqe* complimentary foods are protein-based foods such as offal meats and chicken. However, not all people in the rural community can access these complimentary foods [11]. The sole consumption of wheat-based food products including *Ujeqe* without fruits and vegetables is limited in essential nutrients [10]. Therefore, supplementing cereal-based staple foods such as *Ujeqe* is suggested as a vital medium for essential nutrient delivery, especially for the nutrition security of regular consumers of inadequate foods including *Ujeqe*. Previous studies maintain that Amaranthus is a C4 fast-growing plant and is widely distributed across the world. Amaranthus belongs to the family Amaranthaceae having about 70 species of Amaranthus, of which 17 are grown for edible leaves and 3 are cultivated as food grains [12,13]. However, a few genera of the Amaranthus are used as a traditional medicine; hence, studies show that Amaranthus is an economic and sustainable source of plants with essential nutrients for food and nutrition security, which considers the quality of foods and not just the quantity that is being ingested [14]. Although Amaranthus is a neglected plant, it has been rediscovered as one of the most promising plant genera that contains essential nutrients with phytochemicals, bioactive compounds, and various other valuable nutrients [10,12]. The grain of Amaranthus has been used for the fortification of staple foods, but the leaves have often been neglected as supplements or fortificants [15]. Hence, there is scarce information on the use of the most available underutilized green leafy vegetables such as Amaranthus as a nutrient enrichment especially in *Ujeqe*. This study, therefore, investigates (*Amaranthus dubius*) as nutrient enhancer and sustainable food ingredient to alleviate food and nutrition insecurity of persons in malnourished regions [15]. Cereal-based food improvements have been explored via fortification with nutrient-dense plants such as moringa leaves, seeds, and other nutrient-dense plant materials that are available and easily accessible and have been investigated in staple food [16,17]. However, Amaranthus leaf powder is scarcely utilized, especially in *Ujeqe* steamed bread; therefore, this study explored an innovative attempt to enrich *Ujeqe,* a traditional steamed bread, for the first time.

## 2. Materials and Methods

### 2.1. Research Design

The aim of this study is to investigate the effects of *Amaranthus dubius* leaf powder on the nutritional composition of *Ujeqe*.

An integrated research design comprising qualitative and quantitative data collection was considered appropriate for this study. A purposive convenient random sampling technique was used for population sampling for the sensory evaluation. About 4 to 6 key informant interviews were conducted according to methods described by Muellmmnn, which have been recommended for face-to-face key informant interviews [18]. Thus, a face-to-face interview was conducted in the study area using six key informants who are regular consumers of *Ujeqe* [18]. During the qualitative study, the key informant interview was employed to have insight into the ingredient and traditional method of preparing *Ujeqe,* which was adopted for this study. An experimental study was explored in a standard food laboratory (Consumer Science department) at the University of Zululand KwaZulu-Natal Province Republic, South Africa. During this investigation, several trials of scaling up and down of the ingredients were explored, and a standardized recipe for both the control sample and Amaranthus leaf powder (ALP)-supplemented prototypes of *Ujeqe* were developed.

### 2.2. Sources of Materials, Equipment Used for the Production of ALP Supplemented Ujeqe

**Figure 1:**
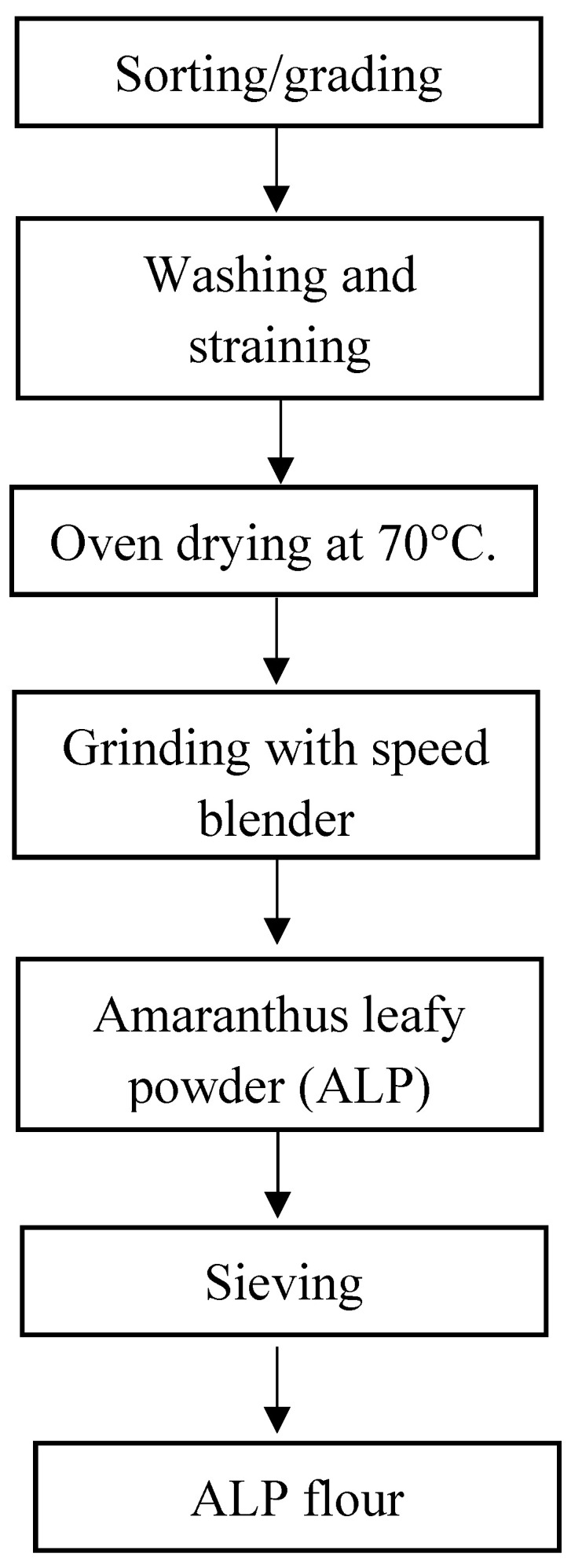
Flow charts for processing of *Amaranthus dubius* leave to Amaranthus leaf powder (ALP).

The *Ujeqe* ingredients for this study were purchased from Shoprite at Esikhawini Empangeni, South Africa. These ingredients include Golden Cloud white bread wheat flour, sunflower oil, Huletts white sugar, Gold Star instant yeast (finest quality), and iodized sea salt. All ingredients were stored separately in an airtight storage box in a refrigerator (4 °C) until they were used. *Amaranthus dubius* leaves were purchased from local vendors at the station market in Empangeni KwaZulu-Natal, South Africa. They were processed into Amaranthus leaf powder (ALP) according to the description in Figure 1.

Similarly, the equipment used in this study includes a Combi-master oven fan, dryer, and electric stove (UK), 0.05 mm sieve, stainless steel colanders, electric blender (Wz-Q10S, Multifunctional speed blender, China), medium stainless steel pot, stainless steel bowls, digital weighing scale, foil paper, aQuelle natural spring still water, ceramic bowls and saucers, serviette, disposable cups, and five-point pictorial smiling face hedonic scale as described in Section 2.11.

### 2.3. Description of the Study Area

‘Empangeni’s geolocation is 28°44′50.3868′ 31°54′42.7078′; it is located 160 km away from Durban along the R34 off the N2, KwaZulu-Natal, South Africa. It is positioned in the hilly countryside of the Uthungulu district with hot, sticky, and languid days. Empangeni’s major crops are cotton, timber, and sugarcane plantations. It is also known for cattle-rearing activities [19]. It was initially the location of the Norwegian Mission station, founded near the stream called Empangeni, which was later moved to Eshowe [19]. The name “Empangeni” comes from a Zulu word called “pangaed”, which means “grabbed”, and it refers to the number of crocodile attacks on water bearers in the nearby Empangeni stream [19].

### 2.4. Production of Amaranthus Leaf Powder

The Amaranthus leaf powder was self-processed, according to Qumbisa (2021) [10] following the procedure in the next subtopic described in the flow chart diagram in Figure 1.

**Figure 2:**
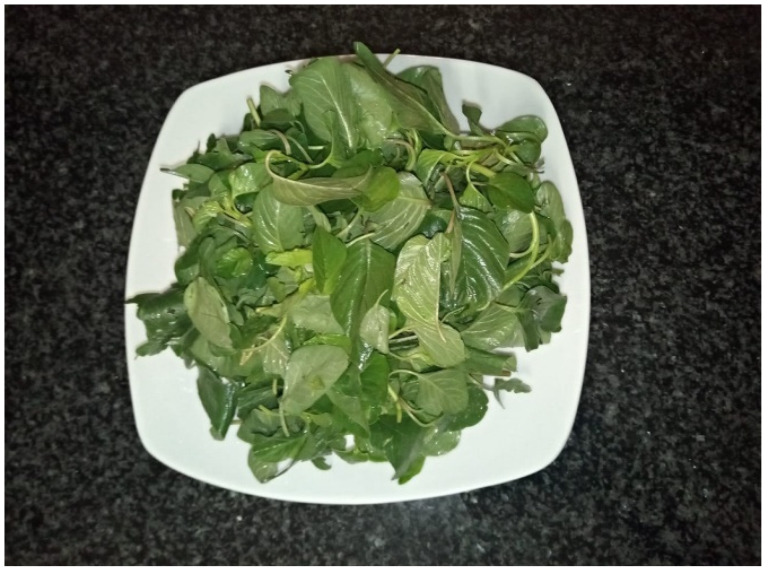
Cleaned *Amaranthus dubius* used for the processing of ALP.

*Amaranthus dubius* leaves described in Figure 2 were sorted, graded, and washed severely under running tap water. This whole process of sorting and grading eliminated the dirt that comes with the Amaranthus leaves. To get rid of contaminants from agricultural processes that may come along with the Amaranthus leaves, two teaspoons of salt were dissolved in 2 L of tap water. They were used to wash the Amaranthus leaves, after which the leaves were rinsed thrice to remove any excess salt [20]. The washed Amaranthus leaves were placed in a strainer for a few minutes to get rid of removal of excess; after the draining of the excess water, the Amaranthus was dried in a combi oven fan dryer at 70 °C for 2 h [20]. The dried leaves were milled into a fine powder using an electric blender (Wz-Q10S, Multifunctional Blender, China). To obtain a uniform Amaranthus leaf powder (ALP), the grounded ALP was sieved through a sieve of 0.05 mm, which was packed into airtight, labeled zip-lock plastic bags until it was used for the production ALP-supplemented *Ujeqe*.

### 2.5. Production of Standardized Amaranthus Leaf Powder Supplemented Ujeqe (ALPSU)

**Figure 3:**
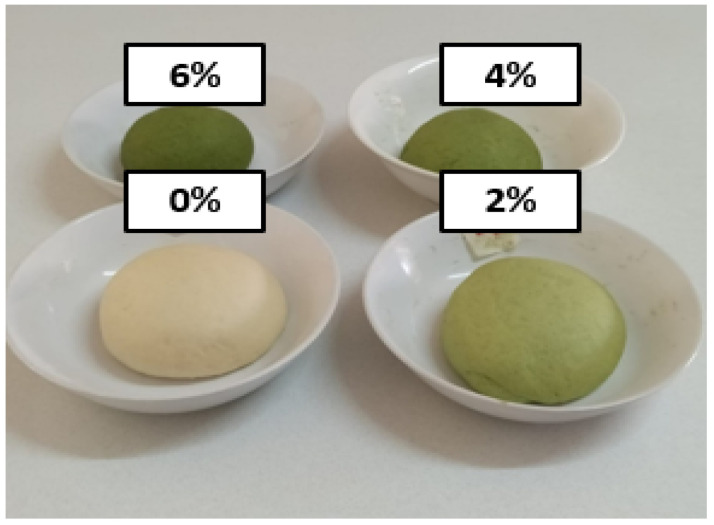
*Ujeqe* dough for the control and for ALP-supplemented prototypes.

An experiment was conducted to establish the effect of ALP on the mineral composition and consumer acceptability of *Ujeqe*. The ingredients in Table 1. were used to formulate a composite flour for ALP-supplemented *Ujeqe* prototypes. Plain wheat flour was substituted for Amaranthus leaf powder (ALP) at ratios of (0%, 2%, 4%, and 6%) and was mixed with other ingredients and bound with lukewarm water to formulate the dough described in Figure 3.

### 2.6. Production of Amaranthus Leaf Powder Supplemented Ujeqe (ALPSU)

**Figure 4:**
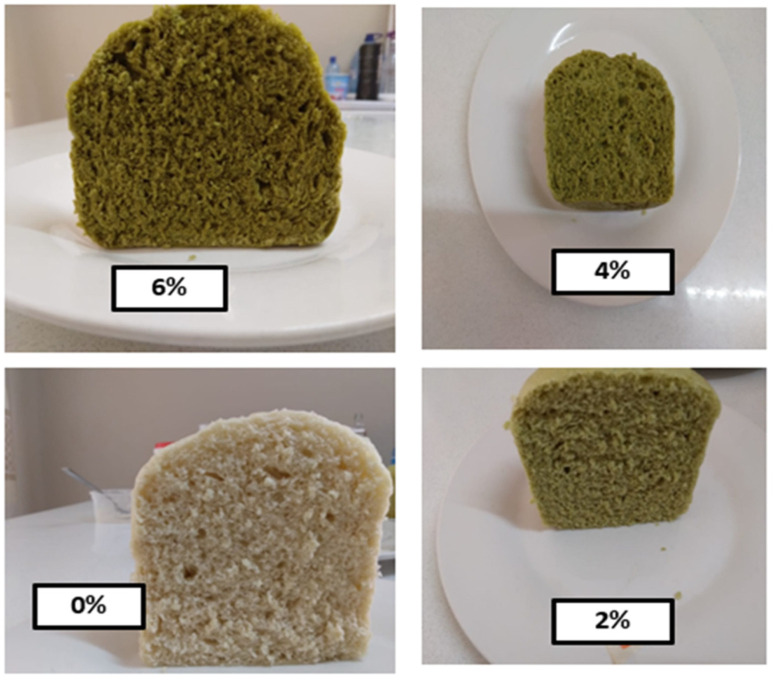
*Ujeqe* ALP 0%, 2%, 4%, and 6% ALP-supplemented *Ujeqe* prototypes.

The control sample (0%, ALP) *Ujeqe* in Table 1 was prepared according to the traditional method adopted from the study site. Likewise, a standard recipe for ALP-supplemented prototypes was achieved after several trials of formulations in a food laboratory consumer science department at the University of Zululand Kwadlangezwa. The standardized recipe for the control sample and the ALP-supplemented *Ujeqe* recipe are described in Table 1. The development of *Ujeqe* food product starts with the formulation of a composite flour next the formulation of the 0% and ALP-supplemented dough formulation, after which plain wheat flour and Amaranth leaf powder were added at an increasing substitution level of 2%, 4%, and 6% ALP). All the dry ingredients of *Ujeqe* acquired were measured in a bowl to obtain a composite flour. Lukewarm water was added to the blended flour to make an elastic, soft, round, molded-shaped dough (6%, 4%, 0%, and 2%) (Figure 3). The dough was manually kneaded for 7 min, after which the dough was covered with a kitchen napkin, and was allowed to ferment (proof) for 40 min. The fermentation or proofing was ascertained when the dough doubled in size. The *Ujeqe* fermented dough was punched to release the in-built air of the dough, after which the dough was molded into a ball again and placed in a stainless bowl used for the steaming of the *Ujeqe.* The stainless bowl was covered with a kitchen napkin and allowed to be proofed a second time for another 40 min. Finally, the stainless bowl was inserted into a large stainless pot of boiling water, and the *Ujeqe* dough was placed into the pot. The dough was not in direct contact with the water. Foil paper was used over the pot before the lid; this is to trap the steam, and the dough was allowed to steam for 50 min on an electric stove. The pot was not opened until after 30 min. A skewer was inserted to check if the *Ujeqe* was cooked, and a well-cooked *Ujeqe* was determined if, when the skewer was inserted, it came out clean. The ALP-supplemented *Ujeqe* samples are shown in Figure 4. ALP *Ujeqe* was kept at room temperature to cool for one hour, after which it was thinly sliced and spread on trays and dried at room temperature [12]. The dried *Ujeqe* was made into a crucible, packaged into a labeled zip-lock bag, and was stored for 48 h before analysis.

### 2.7. Physical Properties of Ujeqe Dough and Ujeqe ALP-Supplemented Food Prototypes

The addition of Amaranthus leaf powder (ALP) to wheat flour led to a change of color of the final dough (Figure 3) from light green to dull green. Additionally, it was observed that an increased concentration of ALP resulted in a lesser elasticity of the dough of the supplemented *Ujeqe* dough. The physical properties of ALP-supplemented *Ujeqe* are described below.

Figure 4 shows the four ALP-supplemented *Ujeqe* prototypes. The increased concentration of ALP in the prototype resulted in a dense ALP-supplemented *Ujeqe.* The density of the *Ujeqe* may be appreciated by older people because they believe that the denser the *Ujeqe,* the more filling it will be. Similar results were reported when the addition of leafy vegetable powder such as moringa oleifera was explored in wheat flour food products. There was a change of color from light green to darker green. This was because of the green pigmentation in the Amaranthus leaves.

### 2.8. Nutritional Analysis of ALP Ujeqe Food Samples

The supplemented *Ujeqe* prototype was dried and processed into a powdered, crucible food material. The raw materials (PWF and ALP), the control food sample (0%), and the three ALP-supplemented prototypes (2%, 4%, and 6%) were packaged in triplicate into an airtight zip-lock plastic bag for nutritional analysis. Following the analysis, in this study, all the proximate content of the food samples (moisture, protein, fat, ash). The content of the crucible food samples was analyzed according to the AOAC Official Method 934.01 (AOAC, 2003) [21]. Whereas the following equation was used to calculate the percentage of protein in the *Ujeqe* prototypes: % crude protein = % N × 6.25. The carbohydrate content in the food samples was determined by difference; that is, (100 minus the total percentage of % protein, % moisture, % fat, % ash). The mineral content of the *Ujeqe* prototypes was determined as ash according to the AOAC official method 942.05 (AOAC, 2003) [21].

### 2.9. Data Analysis

Results obtained from the nutritional analysis presents the average of three replicates, determinations expressed ± as mean and standard deviation (S.D). All the data obtained were statistically analyzed using IBM Corp, released 2020, IBM SPSS statistics for Windows version 27.0 (Armonk, NY, USA, IBM Corp.). Significant difference among the various treatments were evaluated through multivariate analysis of variance (ANOVA), and Duncan multiple comparison post hoc tests were used to separate the means where differences existed (*p* ≤ 0.05).

### 2.10. Ethical Clearance

The ethical clearance for this study was obtained from the Research Ethics Committee at the University of KwaZulu-Natal with reference number HSSREC/00000435/2019. A consent form was used to obtain key informants’ and panelist willingness to participate in the study.

### 2.11. Sensory Evaluation Exercise

A total of N = 60 untrained panelists who are regular consumers of *Ujeqe* were recruited to assess the consumer acceptability level of the ALP-supplemented *Ujeqe* prototypes against the control sample [10,22]. The panelists for the sensory evaluation were chosen because they are regular consumers of *Ujeqe* (steamed bread) and are familiar with and tasted Amaranthus food products. The consent of the panelist was obtained using an informed consent form before the sensory evaluation. *Ujeqe* prototypes were served on a ceramic saucer, and each panelist evaluated four samples comprised of 0%, 2%, 4%, and 6%. Sensory attributes were assessed on a scale of a five-point hedonic scale of pictorial smiling faces, where parameters include dislike very much, dislike slightly, neither liked nor disliked, like slightly, and like very much. The sensory attributes and the overall consumer acceptability level were rated. Panelists were instructed on the right way of filling the scorecard, which evaluates the color, taste, aroma, texture, and overall acceptability. To avoid any biasness, samples were assigned a unique three-digit code; samples were served in a randomized order obtained from a table of random permutations of nine. Additionally, a serviette and a cup of mineral water (aQuelle still water) was provided on the Table of each panelist. Panelists were instructed to rinse their mouths before tasting the first sample of *Ujeqe* and after the testing each of the four *Ujeqe* prototypes. This was carried out to avoid interference with previous taste of any food samples. A serviette was provided on the table of each panelist. Results obtained from all parameters were then statistically analyzed. All analysis was carried out in triplicate, and the data were reported as means ± standard deviation, and significant difference among treatments was evaluated through analysis of variance (ANOVA).

## 3. Results and Discussion

### 3.1. Indigenous Knowledge and Perception of Ujeqe in the Study Area

*Ujeqe* is perceived as a type of traditional bread made by steaming, contrary to the conventional baked white bread consumed worldwide [7]. All groups, excluding babies in the study area, eat *Ujeqe*. *Ujeqe* is a traditional food that is well appreciated because the ingredient is easily accessible and easy to prepare. In the study site, *Ujeqe* is consumed at any time of the day, either as a meal or snack. Furthermore, *Ujeqe* is a traditional food of the Zulus and is a strategic and exceptional food, often served in several conventional events, including weddings and funerals, as well as in cultural and religious ceremonies, especially during ancestral worship [23,24]. Considering the indigenous knowledge system (IKS) perspective, *Ujeqe* has been made from composite flour of mealy maize. However, it is now often made from ingredients such as plain wheat flour (PWF), sugar, salt, and oil or margarine, of which proportions are estimated based on the number of people to be served in the household. Those who are financially buoyant include ingredients such as carrots and milk in their *Ujeqe* recipe. A blend of plain wheat flour and maize flour is believed to make the *Ujeqe* a denser and more filling food for those engaged in strenuous activities. The community members appreciate traditional foods; however, the regular consumption of *Ujeqe* void of nutrient-dense food sources such as fruits and vegetables is considered food that is only high in energy but limited in essential nutrients. Such food can only address the quantity of food, which only caters to the food security of the people and not nutrition security, which considers the adequacy and the quality of food [10]. Food that provides nutrition security is diversified in composition such that it optimizes the inadequacies of an essential nutrient in staple foods, including *Ujeqe* [10,12,23,24].

### 3.2. Macronutrient Composition of the Raw Materials for Ujeqe

Macronutrients are food nutrients that are needed in larger quantities to optimize wellbeing. Macronutrients include carbohydrates, fats, and protein, the essential component of the daily diet that supply energy to humans. The other macronutrients include the food’s fiber, ash, and moisture content. Micronutrients are food nutrients that are essential for wellbeing but are needed in small quantities [25]. The selected macronutrient and mineral content of the raw materials for *Ujeqe* plain wheat flour (PWF) and Amaranthus leaf powder ALP) were analyzed. The results are presented in Table 2 and Table 3.

Table 2 describes the macronutrient composition of plain wheat flour (PWF) and Amaranthus leaf powder (ALP) as the active raw materials that were used to develop *Ujeqe* in this study. The ALP’s ash, fat, and crude protein content were significantly (*p* < 0.05) higher than the content in PWF. Although Amaranthus is a neglected food crop, the proximate content suggests it as a promising supplement for cereal-based food products, including *Ujeqe.*

The moisture content of both raw materials ranged (4.41–10.06%). The study results are similar to trends reported in previous studies [26,27]. The low moisture content of the plain wheat flour and Amaranthus leaf powder are adequate, which implies the keeping shelf-life of the raw materials before it was used for *Ujeqe* production. Amaranthus being a fresh produce is highly perishable, thus either fresh or powdered, it must be well preserved to avoid spoilage and post-harvest losses [28]. Thus, it is identified that Amaranthus processing and preservation must be adequate to prevent quantity and nutrient losses so that it can be available mainly in the lean seasons and at a compensable price [29,30]. Since the moisture of raw materials was low, it implies that high moisture content in the ALP and PWF connotes susceptibility to spoilage, which may lead to the introduction of contaminants in new food products that may lead to the low shelf life of the food product. It was identified that the lower moisture content in cereal flours and leafy greens shows that it was well processed, stored food products [3,27].

Carbohydrate content in this study ranged from 41.6% to 74.03%, and the carbohydrate observed in ALP was significantly (*p* < 0.05) lower compared to that of PWF. It confirms that cereals, including wheat, are higher in carbohydrates than leafy vegetable food materials such as Amaranthus. Thus, the sole consumption of starchy staple foods, including plain wheat-based food products such as *Ujeqe* can result in a high intake of high calories with limited micronutrient intake [31]. Hence, staple foods including cereal foods must be enhanced with nutrient-dense food for optimum nutrition security. The ALP was low in carbohydrates, showing that Amaranthus leaves can complement staple foods; hence, Amaranthus can be utilized to prepare nutritious *Ujeqe* with a low glycemic index.

This study also demonstrates that Amaranthus can be consumed in a quantity that can supply some amount of energy besides its essential nutrient endowment. Amaranthus can be an excellent complementary food for cereal-based food products, including wheat-based products such as *Ujeqe.* Moreover, Amaranthus can be an option of food for a reduced gluten diet, especially for those who are gluten-sensitive or intolerant [12,32]. Additionally, the low content of carbohydrates in this study suggests that Amaranthus leaves are an excellent food for those on a low-carb diet or those that desire to lose weight [33,34,35,36]. The ALP composite flour food products could benefit those with gluten-intolerant problems but also those with casein health challenges/allergies [27,37]. The high ash content observed in ALP compared to PWF suggests there is an appreciable amount of minerals in Amaranthus leaves powder, which can have a positive impact on the nutritional content of the supplemented food products. Table 2 shows that the ash content was higher in ALP than in PWF. A high ash content in food materials, including Amaranthus leafy green, connotes their richness in mineral content. This means that ALP is an excellent source of minerals that can optimize the inadequacies of mineral deficiencies in staple foods.

Similarly, the fat content in this study was higher in ALP than in PWF. The fat in Amaranthus has been identified as trace fats that are free from cholesterol. The high-fat content of the ALP suggests that a blend of any cereal-based foods with ALP would distinctively lead to an enhanced fat content of cereal foods limited in fat. Consequently, Amaranthus’ trace fat content will also lead to the palatability of food products and improved nutrition security [3,38,39]. Likewise, the protein content presented in Table 2 of this study was equally higher in ALP than in PWF, with a statistically significant difference at *p* < 0.05. This study is consistent with a previous study conducted by Famuwagun, (2017) [3], which states that there is high protein content in dried vegetable powders that is evident in ALP; hence, [3].

Table 3 presents the micronutrient composition of (PWF and ALP). Besides potassium, a higher micronutrient content was observed across all the ALP nutritional profile compared to the content in PWF, and the differences recorded were statistically significant at *p* < 0.05. For example, calcium content ranged between (30.00–260.00). Calcium is an essential mineral required for developing strong teeth and bones. It is a vital need in children as well as lactating and pregnant women [40]. The study shows that incorporating ALP in staple foods, including wheat-based *Ujeqe* food products and other calcium-deficient foods, may enhance the calcium status of the final food products.

Similarly, magnesium content ranged from (40.00–110.00), which was higher in ALP than in PWF. The study implies that consuming food with ALP as an active ingredient would enhance the magnesium inadequacies of magnesium in cereal-based foods. Table 3 shows that magnesium is one of the components of Amaranthus, consistent with previous studies [41]. ALP had a higher magnesium content with a statistically significant difference at *p* < 0.05. Green leafy vegetables are excellent sources of magnesium. Magnesium is found throughout the human body; it boosts human performance, combats depression, supports healthy blood sugar levels, and promotes a healthy heart. It has anti-inflammatory benefits that may help prevent migraine attacks and improve premenstrual syndrome. Additionally, the copper content of the supplemented prototype is described in Table 3 and ranges from 1.00–1.73. The sodium content of the raw material in Table 3 is reckoned within the range when compared with the recommended dietary intake for adults (0.02−1.4%) recommended per day; hence, sodium is within the acceptable limits [42]. Low sodium contents in vegetables, including indigenous vegetables such as Amaranthus, have also been reported in previous studies [43].

The iron content in Table 3 ranged from 7.20–24.00. The study shows that ALP had a higher iron composition, implying that Amaranthus leaves are a rich source of iron; therefore, it can be explored as a supplement in cereal-based foods such as *Ujeqe.* Iron-rich foods are fundamental in human nutrition because they have been investigated to play a vital role in the functioning of the human system. For example, iron is essential for transporting oxygen from respiratory organs to other body parts [44]. Iron also prevents anemia and low blood pressure and helps maintain and keep an individual energetic for everyday activities. It is argued that all the essential nutrients in the diet play a crucial role in maintaining an “optimal” immune response. However, insufficient, and excessive intakes can negatively affect the immune status and may render one susceptible to various pathogens. The zinc composition of ALP and PWF ranged from 3.267–7.067; this implies that consumption of ALP food products can provide the consumer with zinc.

Most of the selected minerals analyzed in this study are essential for well-being. All minerals were observed to be higher in ALP composition than PWF; this study suggests that the incorporation of ALP in any staple food may enhance the essential micronutrient inadequacy issues in cereal-based food products, including *Ujeqe* [15]. Amaranthus is suggested as an excellent complement in wheat-based food products, including *Ujeqe.*

Table 3 shows that zinc was higher in ALP than in PWF. This is because zinc is an essential mineral that is needed for the proper functioning of the human system. Although it is considered a trace element, it is vital for human health [41]. It plays a fundamental role in the metabolic processes, while boosting human immunity against diseases and supports the functioning of many biological processes [45]. Low zinc content and ion bioavailability have been linked with limited monoresistance to infections, even during aging [46]. The effects of zinc on human health are based on the zinc ion’s intra- and extracellular regulatory function and its interactions with the proteins. Deficiency of zinc has been linked with impaired metabolic processes, reduced resistance to infections resulting from a poor immune function that may change the skin and its appendages, and disorders of wound healing and hemostasis [41,45]. About 17% of the globe suffers from zinc deficiency. The deficiency of zinc has been known to affect many organ systems and can lead to the dysfunction of many humoral and cell-mediated immunity, thus increasing the susceptibility to infections [41].

Similar to the results of [28], this study also presents a higher copper content in ALP than in PWF. Copper is an essential element for wellbeing; it is required in little amounts by the human body but is beneficial as it helps enzymes transfer energy into the various human cells of the body [47].

The manganese content in this study ranged from 1.43–3.00, and ALP had the highest composition compared to PWF. Manganese is a trace mineral that all humans need in small amounts [47]. However, it is required for the normal functioning of the brain, nervous system, and the body’s enzyme systems. Manganese can be found in seeds and whole grains, as well as in smaller amounts in legumes, beans, nuts, and leafy green vegetables, including Amaranthus [47]. This implies that a diversified diet is key to adequate nutrition for humans’ optimum wellbeing. Furthermore, manganese helps the human body utilize several vitamins, including choline, thiamine, and vitamins C and E, ensuring proper liver function. Additionally, it works as a cofactor, or helper, in development, reproduction, energy production, immune response, and the regulation of brain activity [47].

### 3.3. Effects of ALP Supplementation on Ujeqe Macronutrient Composition

*Ujeqe* is a cereal-based traditional food that is appreciated as a meal and sometimes as a snack in the rural community of Empangeni, South Africa. Since it is a starch-based staple food, it has been identified that monotonous consumption of staple foods, including that of *Ujeqe,* is considered the top contributing cause of micronutrient deficiency challenges across developing countries. Supplementation of cereal-based food has been explored using nutrient-dense materials such as *Moringa oleifera* leaf powder and Amaranthus leaf extract, showing that the food supplements were enhanced nutritionally. The grains of Amaranthus have also been utilized to improve staple food’s nutritional contents. However, the effects of Amaranthus leaf powder supplementation, especially on *Ujeqe* macronutrients, have not been explored [41].

The incorporation of the common *Amaranthus dubius* in the study area enhanced the nutrient content of the supplemented *Ujeqe;* thus Table 4 and Table 5 describe the impact of ALP on both macronutrient and micronutrient composition of the ALP 2%, 4%, and 6% supplemented *Ujeqe* prototype.

Table 4 describes the macronutrient content of the ALP 0%, 2%, 4%, and 6% supplemented *Ujeqe* prototypes. The increased concentration of ALP resulted in a reduced carbohydrate content across all the *Ujeqe* supplemented prototypes but are not statistically significant. A similar decrease in carbohydrates has been reported in a study conducted by [3]. Therefore, the reduction of carbohydrates across supplemented food samples may be attributed to low glycemic index in leafy vegetables including Amaranthus, which has shown that the carbohydrate content in ALP was low. Thus, staple foods containing ALP may have low carbohydrates, which can be an option for those on a low-carb diet. However, the increased ALP in the supplemented prototypes resulted in increased ash content of the *Ujeqe* food prototype, which ranged between 1.39–2.31%. A higher effect of the increased ALP substitution was statistically different at *p* < 0.05. It was observed in Table 4 that the ash content of 2%, 4%, and 6% ALP *Ujeqe* supplemented prototypes were high. This study, therefore, implies that an increased concentration of ALP in staple foods, including *Ujeqe,* enriches the mineral contents of the supplemented prototypes compared to the control sample. This is in agreement with studies that report that foods containing leafy vegetables often have higher ash contents, which connote a mineral-rich food product [4,48]. Table 4 describes the fat content of the ALP-supplemented *Ujeqe* prototype: 4.62–4.96%. Although higher content of fat was reported in 6% substitution of ALP, it was not statistically significant with the control sample and the 4%; it has been reported that leafy vegetables, including ALP, are low in carbohydrates and fats [12,49]. However, the presence of fats in foods enhances the palatability of the food products. Vegetables, including Amaranthus fats, are considered healthy fats because they are considered as unsaturated fat, which includes monounsaturated fat and polyunsaturated fat. It has been reported that fats enhance food taste [3]. It has been reported that green leafy vegetables are sources of essential omega fatty acids, which are exceptionally high in omega-3 compared to omega-6 fatty acids. The presence of these fats in food enhances the palatability and nutritional content of the food products [3]. Since the fat content of the control sample and the ALP-supplemented *Ujeqe* prototype 4 and 4% and 6% was not statistically significant compared to the control, then those desiring to gain weight may need other sources of healthy fats included in their Amaranthus food products to optimize human dietary needs.

Similarly, the protein content of the supplemented prototypes in Table 4 ranged between 13.33–15.56%. A higher increase was recorded in the protein content of the 2% compared with the control sample; similarly, an increase was recorded in 4% ALP-supplemented *Ujeqe* prototypes compared to the control sample and 2% sample. Even though it was there by chance, there was a drop in 6%. However, the 4% and 6% samples in this study demonstrate that a higher substitution of ALP in staple foods can enrich the protein content of any staple-supplemented food products. Table 4 shows that the increased concentration of ALP in *Ujeqe* enhanced the nutritional content of supplemented prototype compared to the control prototype *Ujeqe.* The protein content was observed to be higher in the sample with 2% and 4% ALP substitution. However, the data in 2% and 4% results suggest that the ALP substitution ratios 4 and 6% are not statistically significant. Thus, 2% and 4% in this study, ALP are adequate for improving the macronutrient content of *Ujeqe.* This study indicates that Amaranthus has nutrient-enriching effects on the nutritional composition of the conventional *Ujeqe* and may also enhance similar cereal-based foods with inadequate essential nutrients. The utilization of Amaranthus grains and fortification have been investigated in several studies [12]. However, investigations of ALP in staple foods are scarce/limited. It is essential to note that the information on the application of ALP in *Ujeqe* is scarcely reported; thus, the study appears to be the first to write on the nutrient profile of (0%, 2%, 4%, and 6%) ALP-supplemented *Ujeqe* food products.

### 3.4. Effects of ALP Supplementation on Ujeqe Micronutrient Composition

The effects of legumes, pseudocereals, and leafy vegetables such as moringa oleifera have been used to enhance staple foods for improved food and nutrition security and the alleviation of malnutrition [50,51]. Hence, this study explores the effects of ALP on the micronutrient content of ALP-supplemented *Ujeqe* for improved food and nutrition security.

Table 5 shows that the micronutrient content of ALP-supplemented *Ujeqe* prototypes were enhanced with statistically significant *p* < 0.05. The increase was evident in the following selected minerals, including calcium, potassium, phosphorus, copper, and iron. It has been reported that Amaranthus leaves are dense in essential nutrients, including vitamins and minerals [52]. This study indicates that the increased ALP ratios of ALP in the supplemented prototype resulted in an enhanced *Ujeqe* food product; the enhancement was notable in some highlighted minerals, which are statistically significant *p* < 0.05. It has been identified that both quality and quantity must be considered in meal preparation and consumption. Therefore, to optimize wellbeing, ALP inclusion into other staple foods cannot be overemphasized as this is confirmed to enrich the nutrient content of the staple food products. The *Amaranthus spinous* vegetable variety, including *Amaranthus dubius,* has been reported to have higher nutritional content than other Amaranths species. The ALP-supplemented prototypes were enhanced compared to the control sample; however, not all prototypes were statistically significant. It has been noted that food processing methods such as thermal treatment on leafy vegetables, including Amaranthus, can harm their nutritional content. Most of the heat-sensitive nutrients can be lost or destroyed during food formulation [16,47]. This implied that the nutritional value of *Ujeqe* is dependent not only on the type of species/ingredients used but also on the method of food processing technique and the heat treatment employed. Therefore, the processing of leafy vegetables including Amaranthus should be performed under controlled temperature to minimize nutrient loss.

### 3.5. Sensory Evaluation and Consumer Acceptability of ALP-Supplemented Ujeqe

Table 6 describes the mean and ± standard deviation score for the sensory attributes and the consumer’s overall acceptability of the ALP-supplemented *Ujeqe* prototypes. The color and taste of the ALP-supplemented prototypes were not statistically significant (*p* > 0.05); hence, it did not affect the panelist acceptance of the supplemented samples. The acceptance level of the prototype samples could be because healthy eating is trending worldwide even in South Africa and people are becoming more health-conscious, thus the incorporation of Amaranthus in staple food such as *Ujeqe* was well appreciated among the panelists. Similarly, people in the study area are familiar with the consumption of Amaranthus in its various forms so its color and taste had no negative influence on their acceptance the *Ujeqe* prototypes formulated. This study shows that the control, 0% *Ujeqe* sample from plain wheat flour was highly acceptable by the panelist compared to the three treatments containing ALP at different concentrations (2%, 4%, and 6%). The study further shows that 2% prototype was the most acceptable sample compared to the control sample (0%). However, there was no statistically significant difference between prototype 4% and 6% ALP substitutions. Although aroma, texture, and consumer acceptability were statistically significant at *p* < 0.05. These results are similar to those reported in previous studies [16,20], where leafy vegetables were used in developing staple food products. Amaranthus leaves and their succulent stems are cheap and available. They have been investigated as excellent protein sources with essential amino acids lysine and methionine, carotenoids, ascorbic acid, dietary fiber, and essential minerals, such as calcium, magnesium, potassium, phosphorus, iron, zinc, copper, and manganese [53,54]. In this study, some species of the spinous variety, especially the *Amaranthus dubius* variety, have been recorded as nutrient-dense [22]. It has been widely used in traditional medicine as a medicinal plant to remedy malarial, diabetic, bacterial, and viral diseases, helminthic infections, and as a snake antidote [12,32,49]. *Amaranthus dubius* leaf powder is an excellent sustainable food plant that can be explored in various staple food products for improved food and nutrition security; and addressing micronutrient deficiencies challenges especially among the malnourished population.

## 4. Conclusions

*Amaranthus dubius* leaves were self-processed into Amaranthus leaf powder (ALP) under a controlled food laboratory condition. The raw materials, plain wheat flour (PWF) and ALP, were analyzed in triplicate for nutritional composition. ALP had higher nutritional composition compared to plain wheat flour. The ALP ratio: (0%, 2%, 4%, and 6%) was explored in plain wheat *Ujeqe*, and supplemented *Ujeqe* prototypes were enhanced in macro-nutrients. Notably, the protein content may provide food and nutrition security for protein-malnourished populations. Likewise, the ash content of the ALP-supplemented prototypes was enhanced, which depicts that the enriched prototypes were enriched mineral-wise. Thus, the selected minerals content of the supplemented *Ujeqe* was statistically significant at the *p* < 0.05 level. Zinc is an essential mineral that is needed to optimize wellbeing. Although the zinc content increased across the prototypes, the mean value differences were insignificant. The sensory evaluation result shows that aroma and texture were statistically significant at *p* < 0.05, implying that increased ALP substitution affected the consumer preference for texture and aroma of the prototype samples. However, increased ALP in *Ujeqe* did not affect the consumer acceptability of the color and taste of the supplemented prototypes (Table 6). The findings of this study show that increase in ALP supplementation did not compromise the overall acceptability of *Ujeqe* prototypes. The 6% ALP *Ujeqe* was nutritionally enhanced; however, it was the least appreciated sample, but there was no statistical significance between the prototype 4% and the 6% sample. However, the 2% ALP *Ujeqe* prototype was the most acceptable in this study compared to the reference (control) 0% sample. Amaranthus is one of the cheapest, most nutrient-dense underutilized leafy vegetables with excellent potential to optimize the dietary needs of the malnourished population with limited resources. There is a need to disseminate information on the nutritional prospect of ALP-supplemented food products such as ALP-supplemented *Ujeqe* via workshops, seminars, or campaigns. Food-based strategies encouraging a diversified diet, such as ALP supplementation in staple foods, are tools for tackling malnutrition at the household level. Additionally, fiber is one of the components of leafy vegetables, including Amaranthus; however, the fiber content was not analyzed in this study. Hence, future studies can include fiber analysis and shelf-life study of ALP *Ujeqe* samples.

## Figures and Tables

**Table 1:** Recipe for wheat-based ALP-supplemented *Ujeqe*.

Amaranthus %	PWF (g)	Yeast (g)	Sugar (g)	Salt (g)	Sunflower Oil mL	Water mL
0	100	3	10	1	7.5	60
2	98	3	10	1	7.5	60
4	96	3	10	1	7.5	60
6	94	3	10	1	7.5	60

Amaranthus leaf powder (ALP), Plain wheat flour PWF.

**Table 2:** Macronutrient composition of wheat flour and Amaranthus leaf powder.

(Macronutrient (g/100 g Dry Matter Basis)	PWF	ALP
Carbohydrate	74.03 ± 2.21 ^a^	41.6 ± 0.61 ^b^
Moisture	10.06 ± 0.08 ^a^	4.41 ± 0.34 ^b^
Ash	2.37 ± 0.12 ^a^	17.97 ± 0.11 ^b^
Fat	1.58 ± 0.14 ^a^	4.47 ± 1.32 ^b^
Protein	11.96 ± 2.31 ^a^	31.56 ± 1.76 ^b^

Mean values ± SD with the different superscripts across the row are significantly (*p* ≤ 0.05) different. PWF means plain wheat flour, and ALP means Amaranthus leaf powder.

**Table 3:** The mineral content of wheat flour and Amaranthus leaf powder.

(mg/100 g Dry Matter Basis)	(PWF)	(ALP)
Calcium	30.00 ± 0.00 ^a^	2600.00 ± 0.03 ^b^
Magnesium	40.00 ± 0.00 ^a^	1210.00 ± 0.01 ^b^
Potassium	5400.00 ± 0.11 ^a^	160.00 ± 0.1 ^b^
Sodium	60.00 ± 0.01 ^a^	130.00 ± 0.01 ^b^
K/Ca^+^ Mg	0.11 ± 0.00 ^a^	0.05 ± 0.00 ^b^
Phosphorus	0.02± 0.00 ^a^	0.06 ± 0.01 ^b^
Zinc	3.27 ± 0.58 ^a^	7.07 ± 0.58 ^b^
Manganese	1.43 ± 0.58 ^a^	3.00 ± 0.00 ^b^
Copper	1.00 ± 0.00 ^a^	17.34 ± 0.58 ^b^
Iron	7.20 ± 3.61 ^a^	24.00 ± 16.83 ^b^

Mean values ± SD; the different superscripts across the row are significantly different at *p* < 0.05. PWF means plain wheat flour, and ALP means Amaranthus leaf powder.

**Table 4:** Macronutrient composition of ALP-supplemented *Ujeqe* prototypes.

(ALP %)				
(g/100 g Dry Matter Basis)	0	2	4	6
Carbohydrate	73.21 ± 0.25 ^b^	71.09 ± 0.45 ^a^	70.32 ± 0.31 ^a^	70.26 ± 0.56 ^a^
Moisture	7.49 ± 0.14 ^a^	8.03 ± 0.03 ^d^	7.67 ± 0.05 ^b^	7.85 ± 0.1 ^c^
Ash	1.39 ± 0.03 ^a^	2.2 ± 0.01 ^c^	2.05 ± 0.01 ^b^	2.31 ± 0.03 ^d^
Fat	4.62 ± 0.13 ^b^	3.39 ± 0.15 ^a^	4.4 ± 0.42 ^b^	4.96 ± 0.49 ^b^
Protein	13.33 ± 0.02 ^a^	15.27 ± 0.33 ^b^	15.56 ± 0.65 ^b^	14.64 ± 0.5 ^b^
Energy (Kcal)	387.58 ± 0.48 ^b^	376.04 ± 0.87 ^a^	381.08 ± 3.87 ^ab^	380.15 ± 2.61 ^a^

Mean values ± SD with the different superscripts across the row are significantly different at *p* < 0.05. ALP means Amaranthus leaf powder.

**Table 5:** The mineral content of ALP-supplemented *Ujeqe*.

		(ALP %)		
(mg/100 g Dry Matter Basis)	0	2	4	6
Calcium	30.00± 0.00 ^a^	70.00 ± 0.00 ^b^	120.00 ± 0.01 ^c^	140.00 ± 0.01 ^d^
Magnesium	120.00± 0.12 ^a^	50.00 ± 0.01 ^a^	1 0.00 ± 0.00 ^a^	80.00 ± 0.04 ^a^
Potassium	210.00 ± 0.00 ^a^	280.00 ± 0.01 ^b^	370.00 ± 0.01 ^c^	430.00 ± 0.02 ^d^
Sodium	290.00 ± 0.00 ^a^	520.00 ± 0.00 ^c^	300.00 ± 0.00 ^a^	360.00 ± 0.06 ^b^
K/Ca^+^ Mg	0.07 ± 0.42 ^a^	0.08 ± 0.02 ^a^	0.07 ± 0.02 ^a^	0.08 ± 0.20 ^a^
Phosphorus	0.01 ± 0.00 ^a^	0.03± 0.07 ^b^	0.02± 0.00 ^a^	0.02 ± 0.20 ^a^
Zinc	3.27 ± 2.08 ^a^	3.20 ± 0.00 ^a^	3.3 7± 0.58 ^a^	3.47 ± 3.06 ^a^
Manganese	0.80 ± 0.00 ^a^	0.90 ± 0.00 ^a^	1.00 ± 0.00 ^a^	1.00± 2.65 ^a^
Copper	0.17 ± 0.58 ^a^	0.17 ± 0.57 ^a^	0.30± 0.00 ^b^	0.2 7 ± 0.58 ^ab^
Iron	5.17 ± 7.40 ^a^	5.27 ± 2.08 ^a^	6.13 ± 2.31 ^b^	6.50 ± 14.18 ^c^

Mean values ± SD with the different superscripts across the row are significantly different at *p* < 0.05. Amaranthus leaf powder.

**Table 6:** Sensory evaluation and consumer acceptability of ALP-supplemented *Ujeqe*.

	ALP Composition (%)			
Sample	0	2	4	6
Color	3.00 ± 0.00 ^a^	3.00 ± 0.00 ^a^	3.00 ± 0.00 ^a^	3.00 ± 0.00 ^a^
Aroma	4.55 ± 0.7 ^abc^	4.12 ± 0.87 ^ad^	3.9 ± 0.84 ^bc^	3.44 ± 1.1 ^cde^
Taste	4.00 ± 0.00 ^a^	4.00 ± 0.00 ^a^	4.00 ± 0.00 ^a^	4.00 ± 0.00 ^a^
Texture	4.90 ±0.31 ^abc^	4.52 ± 0.63 ^ad^	4.24 ± 0.86 ^be^	3.59 ± 1.14 ^cde^
Overall Acceptability	4.82 ± 0.4 ^ab^	4.49 ± 0.71 ^c^	4.24 ± 0.88 ^ad^	3.59 ± 1.13 ^cd^

Mean values ± SD with the different superscripts across the row are significantly different at *p* < 0.05. Amaranthus leaf powder (ALP).

## Data Availability

Data is contained within the article.
